# Plant Pollinator Networks along a Gradient of Urbanisation

**DOI:** 10.1371/journal.pone.0063421

**Published:** 2013-05-22

**Authors:** Benoît Geslin, Benoit Gauzens, Elisa Thébault, Isabelle Dajoz

**Affiliations:** 1 Laboratoire Biogéochimie et Écologie des Milieux Continentaux UMR 7618, Centre National de la Recherche Scientifique, Paris, Île-de-France, France; 2 Université Paris Diderot, Paris, Île-de-France, France; 3 Université Pierre et Marie Curie, Paris, Île-de-France, France; 4 École Normale Supérieure, Paris, Île-de-France, France; University of Northampton, United Kingdom

## Abstract

**Background:**

Habitat loss is one of the principal causes of the current pollinator decline. With agricultural intensification, increasing urbanisation is among the main drivers of habitat loss. Consequently studies focusing on pollinator community structure along urbanisation gradients have increased in recent years. However, few studies have investigated how urbanisation affects plant-pollinator interaction networks. Here we assessed modifications of plant-pollinator interactions along an urbanisation gradient based on the study of their morphological relationships.

**Methodology/Principal Findings:**

Along an urbanisation gradient comprising four types of landscape contexts (semi-natural, agricultural, suburban, urban), we set up experimental plant communities containing two plant functional groups differing in their morphological traits (“open flowers” and “tubular flowers”). Insect visitations on these communities were recorded to build plant-pollinator networks. A total of 17 857 interactions were recorded between experimental plant communities and flower-visitors. The number of interactions performed by flower-visitors was significantly lower in urban landscape context than in semi-natural and agricultural ones. In particular, insects such as Syrphidae and solitary bees that mostly visited the open flower functional group were significantly impacted by urbanisation, which was not the case for bumblebees. Urbanisation also impacted the generalism of flower-visitors and we detected higher interaction evenness in urban landscape context than in agricultural and suburban ones. Finally, in urban context, these modifications lowered the potential reproductive success of the open flowers functional group.

**Conclusions/Significance:**

Our findings show that open flower plant species and their specific flower-visitors are especially sensitive to increasing urbanisation. These results provide new clues to improve conservation measures within urbanised areas in favour of specialist flower-visitors. To complete this functional approach, studies using networks resolved to the species level along urbanised gradients would be required.

## Introduction

One of the major causes of the observed pollinator decline [1] is the loss of natural environments through habitat modification [2]. Increasing urbanisation is an important driver of this habitat alteration [3], with the expansion of impervious areas at the cost of natural habitats [4]. The number of studies targeting the impact of urban areas on pollinating fauna has increased in the past few years, most of them focusing on the description of abundance and diversity of urban pollinators [4]–[9]). The negative effects of urbanisation on pollinator communities are likely to impact plant-pollinator interaction networks [10] and consequently the reproductive success of plant communities. This is topical in pollination ecology, since a loss of species leads to a loss of interactions which in turn causes a loss of functions, and these interactions among organisms are key providers of ecosystem services [11].

At a microcosm scale of thirty plants, it has been experimentally demonstrated that a loss of functional diversity within a pollinator community could impair the reproductive success and the persistence of a plant community [12]. On a larger scale, Biesmeijer *et al*. [13] documented important parallel decline of flower specialist pollinators and their obligatory insect-pollinated plants across two European countries, also suggesting a strong link between plant and pollinator community dynamics. The studies of Fontaine *et al.* [12] and Biesmeijer *et al*. [13] underline the importance of studying plant-pollinator networks to understand mechanistically the links between plant and pollinator communities and the consequence of species loss.

These studies also both highlight the importance of considering plant and pollinator functional traits in this context (see also [14], [15]). Pollinators with narrow habitat requirements and low mobility tend to decline more than generalist and mobile species ([13], [16]; see also [8]). In a plant community containing tubular flowers, Fontaine *et al*. [12] also showed that the reproductive success was lowered when the pollinating fauna was only composed with short mouthparts species compared to a pollinating fauna composed with short and long mouthparts. Recent studies on the relationship between diversity and ecosystem functioning indicate that the functional diversity of traits matters more than species richness [17], [18]. Understanding consequences of pollinator decline on plant communities should thus couple a plant-pollinator network approach and a focus on species functional groups.

To date, we still ignore how changes in pollinating fauna induced by urbanisation could impact the structure of plant-pollinator networks (but see [19]) and the functioning of plant communities. Here we focused on an urbanisation gradient in the Ile-de-France region (an area of 12 000 km^2^ around Paris, France) which encompassed four types of habitats, ranging from semi-natural and agricultural landscapes to suburban and densely urbanised (Paris) areas. We tested how modifications in pollinator community structure resulting from landscape variations along urbanisation gradients may affect plant communities. To do so, we set up an experimental plant community comprising two plant functional groups based on corolla morphological traits (“open flowers” with easily accessible floral rewards and “tubular flowers” with hidden rewards for short mouthparts pollinators; see [12]), and replicated these communities along our urbanisation gradient. Then, we built quantitative plant-pollinator networks based on flower visitors identified at the morphotype or morphospecies level [20].

By analysing the topology of these interaction networks (arrangement and connectivity), we aimed to assess (i) if, and how, the number of interactions performed by the different pollinator morphological groups and their pollinating behaviour would be modified along an urbanisation gradient, (ii) how these modifications could impact on the structure of the plant-pollinator networks and (iii) how this could in turn affect the potential reproductive success of our experimental plant community. Our results show that increasing urbanisation leads to a decline of pollinators visiting open flowers (short mouthparts morphological group) and an increase of their generalism. These results in turn cause a decrease of the potential reproductive success of the open flowers functional group.

## Materials and Methods

### Study Sites

**Figure 1:**
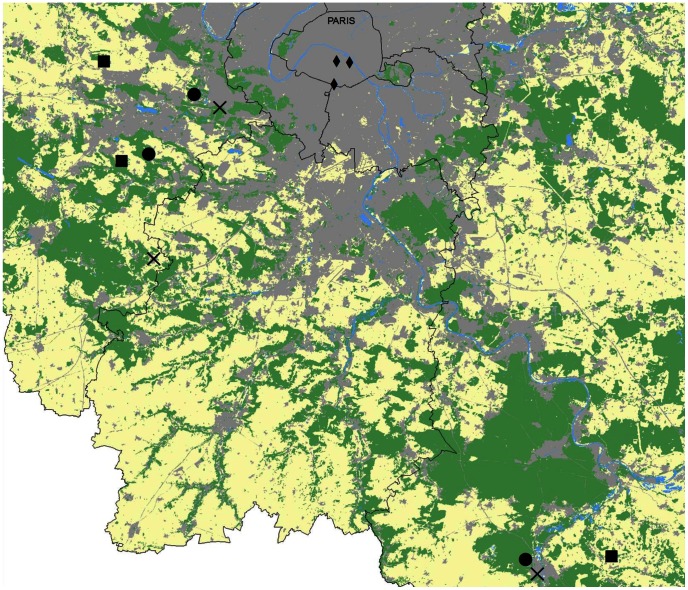
Distribution of agricultural (squares), semi-natural (dots), suburban (crosses) and urban (diamonds) sites. Land Cover data were obtained with the permission of the IAU Île-de-France, MOS 2008. Base map colours represent: areas dominated by agricultural landscape (yellow), by semi-natural habitats (green) or by impervious zones (grey). Water-covered surfaces are represented in blue.

The administrative region of Paris (Ile-de-France, France, Fig. 1) shows a great diversity of ecosystems ranging from semi-natural grasslands and agricultural landscapes to densely populated urban areas. In this region, we focused on four landscape contexts: semi-natural, agricultural, suburban and urban. Within each landscape context, three experimental sites were selected (see Fig. 1). These twelve experimental study sites were chosen according to their land cover within a 500 meter radius. We used Geographic Information Systems (GIS, ESRI ARC INFO v. 10.0) to estimate the proportion of several habitat types within each of the twelve study sites. In semi-natural sites, more than 50% of the area was covered by forest and permanent grassland. Agricultural sites were covered by more than 40% of arable land. Suburban sites were covered by 25% to 50% of impervious areas, and urban sites were characterized by more than 50% of impervious areas.

### Plant Community

An experimental plant community was established in each of the twelve sites. This community comprised five entomogamous species distributed between two functional groups based on corolla morphological traits. In the “tubular flowers” functional group, rewards to pollinators (i.e. pollen and nectar) are set within the corolla tube and are difficult to access for pollinators with short mouthparts [12]. This group comprised the three following species: *Lotus corniculatus* L., *Medicago sativa* L. and *Consolida regalis* Gray. The “open flowers” functional group comprised two species: *Matricaria inodora* L. and *Sinapis arvensis* L., both species with rewards on top of a flat corolla, easily accessible to pollinators with short and long mouthparts [12]. Seeds of these five species (Baumaux, Nancy, France) were germinated and grown for two months (February–March 2011) in greenhouse conditions (sodium lighting during 14 hours per day) before transplantation in the field (April 2011). These five species are visited by pollinators in natural conditions. *L. corniculatus* is a self-incompatible species [21] such as *S. arvensis* [22], *M. inodora* [23] and *C. regalis* [24]. Self-fertilization could happen in *M. sativa* but this species is mainly allogamous [25]. Moreover all these species are native of the Ile-de-France region [26].

### Spatial Configuration of Plant Communities

Four plots (1.80 m*2.10 m) containing 6 individuals of each plant species (for a total of 30 plants per plot) were set up in each of the twelve sites. In these plots, plants were spaced following a grid of 30 square cm. Plots were designed following two spatial configurations. In half of the plots, plants were set up following a systematic configuration with each individual per species planted in a row. In the two other plots, plants were set up following random configuration of individuals, and the same random arrangement was used in all plots designed under this configuration. The flowering quality of the plant community was estimated throughout the experiment. Before each observation session, all plants of each species were surveyed. We built an indicator ranging from 0 to 6 regarding the number of flowering plants per species. This indicator was used to control for potential variations in flowering quality between experimental sites in statistical tests (see below) as flowering quality of plants is susceptible to affect pollinator visitation frequency [27].

### Recorded Data

During the peak flowering period, from early May to end of June 2011, 4 observation rounds (one every two weeks) were carried out on each site. During these rounds, all flower–visiting insects foraging on the experimental plant communities were recorded during 10 minutes within each patch in order to build plant-pollinator networks. For each visit, the identity of the visited plant species was recorded. Numbers of observed visits were used as surrogate for the strength of interactions. Pollinator observations were always carried out during sunny, not windy days between 10 am and 5 pm to minimize variations due to climatic conditions. In order to use a non-intrusive method, pollinators were only observed in the field and classified into morphological functional groups. Even though identifying insects to the species level would have been ideal, the difficulty to identify pollinators to the species level under field conditions [28], [29] prompted us to identify insects to the morphotype level.

### Insect Functional Morphotypes

The nine morphotypes we studied were: 1) “Bumblebees” (group including all individuals belonging to the Bombus genus). 2) “solitary bees” (group enclosing bees from the Apoidea super-family except those from genus Bombus, *Apis mellifera*, and Sphecidae. This group included individuals from the following families: Andrenidae, Colletidae, Dasypodaidae, Halictidae, Melittidae, Apidae and Megachilidae families). Most of the individuals we observed belonged to the Halictini tribe (unpublished data). 3) “*Apis mellifera*” (group comprising individuals of *Apis mellifera*). 4) “Coleoptera” (group including all beetles which were feeding on our plant communities). 5) “Lepidoptera” (group including all butterfly individuals). 6) “Syrphidae” (group including all individuals from the Syrphidae family (Diptera)). 7) “Other flies” (group including all non-Syrphidae Diptera). 8) “Other Hymenoptera” (group including aculeate wasps belonging to the Vespidae family). And 9) “bugs” (group gathering all Heteroptera which were feeding on our plant communities).

### Data Analysis

#### Interaction matrices

To compute network metrics, pollination webs were represented as matrices, with n lines (representing the plant species described above), and *m* columns (representing the insect morphotypes described above). The value of A_i,j_ (the intersection of the *i* ^th^ line with the *j* ^th^ column in matrix A) represents the number of interactions observed between plant *i* and insect morphotype *j*. In order to remove the potential effects of asynchrony in plants flowering, we considered networks cumulated over time. In total, we obtained 24 webs (one for each plant spatial configuration in each observation site) representing all interactions occurring during all observation rounds. All the following descriptors were computed using the Rpackage bipartite [30].

#### Network interaction diversity (see [31])

To determine the diversity of interactions in each of the four types of networks obtained (in the semi-natural, agricultural, suburban and urban landscape contexts respectively) we used the *interaction evenness* (the same method was used in [32]), whose calculation is similar to the Shannon index, but this parameter does not take into account the absence of pollination links (i.e. network matrices entries equal to zero) for its calculation. The interaction evenness reaches 1 when the number of interactions between insect morphotypes and plants species is uniformly distributed, showing a homogeneous distribution of interactions within networks.

#### Species generalism

Pollinator generalism was estimated using two methods. First, we calculated a qualitative measure of generalism, defined as the number of plants with which a given morphotype of flower visitors interacts in the network. The second measure took into account the link intensity (i.e. number of interactions for each link) and it was computed with the Shannon index.

#### Plant reproductive success index

We developed an index to estimate the actual number of visits that could potentially affect the reproductive success of the considered plant species. To be pollinated, plants need to receive pollen from a plant of the same species. Here we considered that only visiting flowers of a same species in a row (*i.e.* flower constancy), provides fitness benefits by facilitating transfer of pollen between conspecific plants [33], [34]. Indeed, it has been shown that pollinators visiting flowers of different species may either loose pollen during interspecific flights or even induce cluttering of stigmas with foreign pollen after interspecific visits [35], [36]. Thus we considered that a visitation event impacted on the reproductive success of species when this plant species was visited twice in a row by the same insect. The probability for each pollinator *p_i_* to visit a plant species *f_i_ i*s *P_pi_,_fi_,* defined as the number of observed interactions of the pollinator group *p_i_* on *f_i_* divided by the total (for all plants) number of interactions carried out by the pollinator group *p_i_. T*he probability for insect *p_i_* to visit twice in a row the plant *f_i_* is *P^2^_pi,fi_*. For a given plant species, the expected number of reproductive events provided by pollinator *p_i_* is thus P^2^ *_pi,fi_* multiplied by the number of individuals of *p_i_* observed, which are estimated here by the number of observed visits *V_i_* performed by pollinator *p_i_*.

The reproductive success index of a plant species is then the sum of reproductive events carried out by the *n_p_* flower visitor morphotypes observed foraging on this plant species, with a probability *P_i fi_*:
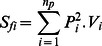



The reproductive success index per plant functional group was estimated as the sum of the reproductive successes of all plants belonging to this functional group.

#### Statistical analyses

All statistical analyses were performed with R 2.14.0 software [37]. First, we checked for potential spatial autocorrelation in our data. To do so, we calculated the Bray-Curtis similarity index on the number of interactions performed by flower visitors on experimental plant communities in all pairs of sites [38]. The geographical distance between all pairs of sites was also calculated. The resulting geographical and similarity distance matrices were used to test for spatial autocorrelation with a Mantel test (999 permutations; same method was used in [8]). There was no significant spatial autocorrelation (*P = *0.54) among the sites in each of our four landscape contexts.

We performed generalized linear mixed effect models (GLMM; [39]) to analyse the effect of landscape context on the number of interactions at network level and morphotype level, and on the number of links per networks. As numbers of interactions were count data we fitted models with a Poisson distribution and a log link. Fixed effects were the landscape context and the flowering quality of plant communities (and the identity of the insect morphotype for analyses at the morphotype level). Random effects were the experimental site and the plant spatial configuration, to avoid pseudo-replication. Models were simplified by backward selection based on AIC (drop1 function). Significance of fixed effects and their interactions were tested by comparing models with a likelihood-ratio test (i.e. Chi squared test, see [40]).

To analyse the effect of landscape context on the evenness index, the weighted generalism degree of insect morphotypes and the reproductive success index of plant morphotypes, we used linear mixed models with the lme function from the nlme package [41], [42]. Fixed effects were the landscape context and the flowering quality of plant communities (and the identity of the insect morphotype for analyses of the generalism degree of insect morphotype; and the identity of plant morphological group for analyses of the reproductive success index of plant morphotypes). Random effects were the experimental site and the plant spatial configuration to avoid pseudo-replication. Histograms of models residuals were plotted to check for normality as suggested by Zuur et al. [43]. Parameters of statistical models were estimated using maximum likelihood. Models were simplified by backward selection based on AIC (drop1 function). Significance of fixed effects and their interactions were tested by comparing models with a likelihood-ratio test (i.e. Chi squared test).

Post-hoc test were performed using glht function of the package multcomp [44]. To discriminate between all pairs of treatments, they were compared with Tukey’s test.

We used Chi-squared analyses to analyse the distribution of interactions performed by each insect morphotype among the four landscape contexts.

## Results

### Overall Characteristics of Pollination Networks

**Figure 2:**
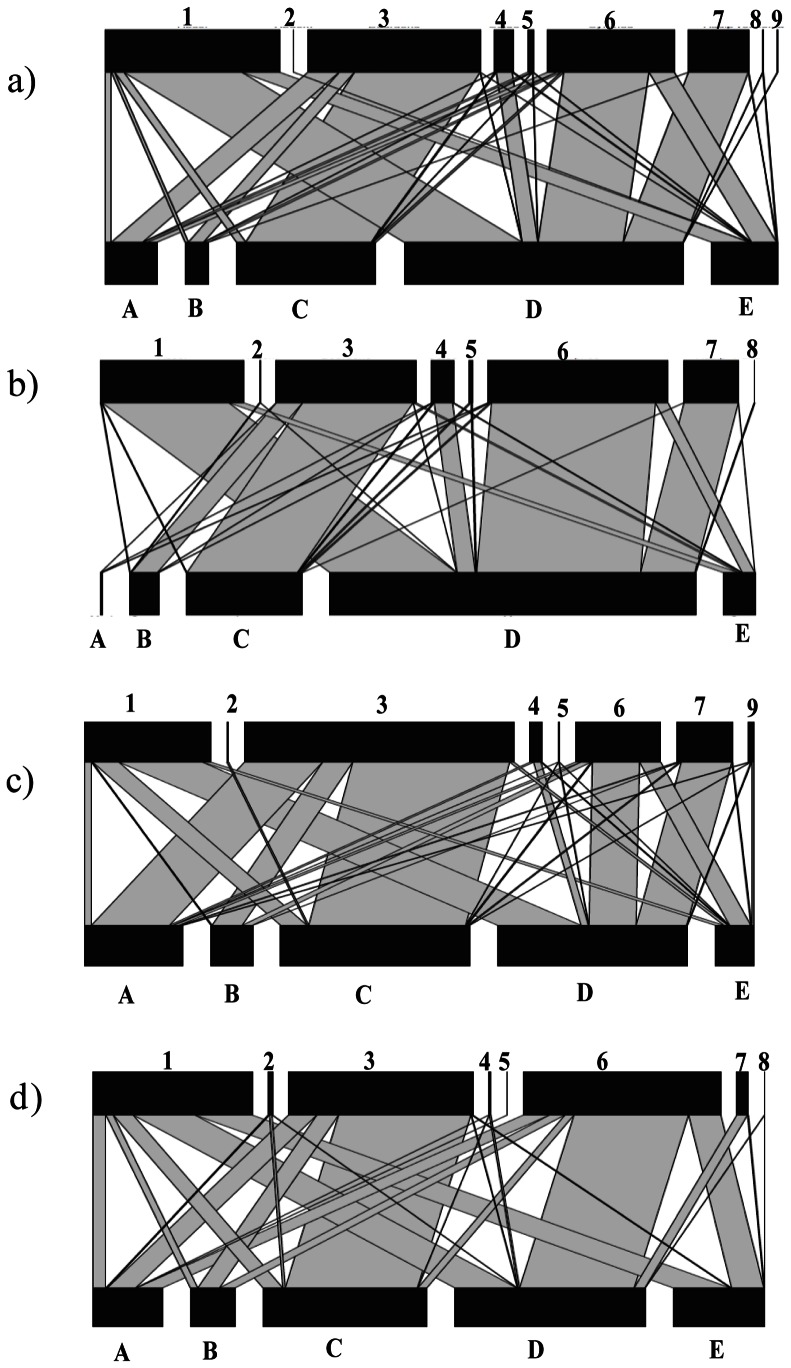
Flower-visitor interaction network structure in the different landscape contexts. a) Semi-natural network, b) Agricultural network, c) Suburban network, d) Urban network. The width of the links is proportional to the number of visits observed. 1: solitary bees; 2: *Apis mellifera*; 3: bumblebees; 4: Coleoptera; 5: Lepidoptera; 6: Syrphidae; 7: other flies; 8: bugs; 9: Vespoidae. A: *Medicago sativa*; B: *Consolida regalis*; C: *Lotus corniculatus*; D: *Matricaria inodora*; E: *Sinapis arvensis*.

We observed 17 857 interactions between the nine morphotypes of floral visitors and the five plant species of our experimental plant community, in all twelve sites over the course of the experiment. Cumulative networks of these interactions for each landscape are presented in Fig. 2. Bumblebees were involved in 31.1% of interactions (5 567 interactions); solitary bees in 28.2% (5 038 interactions); *Apis mellifera* in 0.2% (36 interactions); Coleoptera in 3.1% (565 interactions); Lepidoptera in 0.67% (118 interactions); Syrphidae in 27% (4 789 interactions); other flies in 9.4% (1 686 interactions); bugs in 0.18% (32 interactions) and other Hymenoptera in 0.15% (26 interactions).

### Effects of Urbanisation on Plant-pollinator Networks

**Figure 3:**
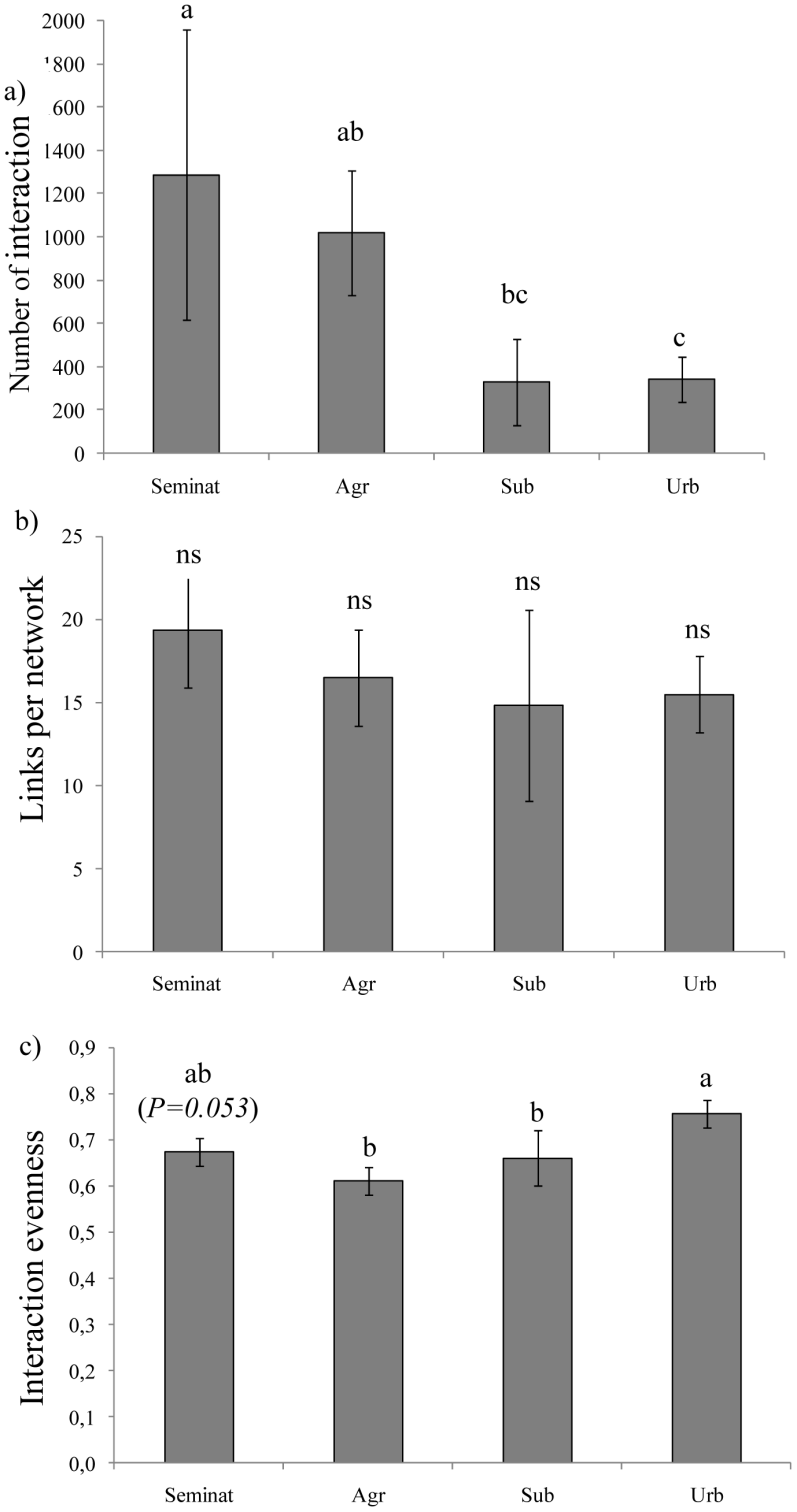
Network level indices pollinators according to the landscape context. a) Mean number of interactions between plants and flower-visitors b) Mean number of links per network. c) Mean interaction evenness. Lines above bars denote 95% confidence interval. Bars that do not share the same letter show significant differences (P<0.05). NS: no significant differences (P>0.05). Seminat: Semi-natural; Agr: Agricultural; Sub: Suburban; Urb: Urban.

The mean number of insect visits per minute and per landscape on experimental plant communities was respectively: 16.10±4.29 (semi-natural context), 12.73±1.82 (agricultural), 4.08±1.27 (suburban) and 4.23±0.67 (urban). Landscape context significantly influenced the mean number of interactions per network (Fig. 3a; *X^2^*_3_ = 8.09, *P = 0.044*) whereas we did not detect any impact of the landscape context on the mean number of links per networks (Fig. 3b; *X^2^*_3_ = 1.72, *P = 0.63*). More precisely, the mean number of interactions in semi-natural landscape networks was significantly higher than in urban (Tukey test *P<0.001*) and suburban networks (Tukey test *P = 0.009*), and the mean number of interactions in agricultural networks was significantly higher than in urban ones (Tukey test *P = 0.03*). The difference in the mean number of interactions between agricultural and suburban networks was not significant (Tukey test *P = 0.21*).

Landscape context significantly influenced the interaction evenness (Fig. 3c; *X^2^*_3_ = 11.47, *P = 0.009*). In urban landscape networks, the mean interaction evenness was significantly higher than in suburban (Tukey test *P = 0.008*) and agricultural landscape networks (Tukey test *P<0.001*). The difference between urban networks and semi-natural landscape networks was marginally non-significant (Tukey test *P = 0.053*).

### Effects of Urbanisation on Plant and Flower-visitor Morphotypes

#### Flower visitors functional groups

**Figure 4:**
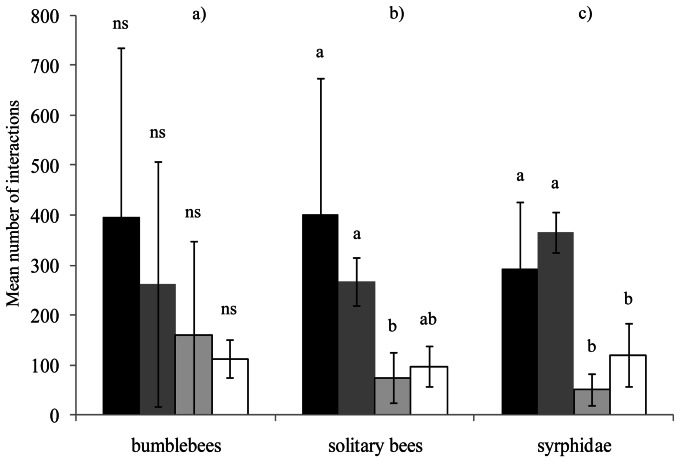
Mean number of flower interactions according to the landscape context. a) bumblebees. b) solitary bees c) Syrphidae. Black bars: semi-natural context; dark grey bars: agricultural context; light grey bars: suburban context; white bars: urban context. Lines above bars denote 95% confidence interval. Bars that do not share the same letter show significant differences (P<0.05). NS: no significant differences (P>0.05).

Number of interactions: Regarding flower visitors morphotypes, the effect of the urbanisation gradient depended upon the morphotype considered (Table 1; significant interaction between insect morphotypes and landscape context *X^2^*_24_ = 1026.6, *P<0.001*). While we did not detect any effect of the landscape context on the number of interactions performed by bumblebees (Fig. 4a; *X^2^*_3_ = 2.76, *P = 0.42*), other Hymenoptera (marginally non-significant *X^2^*_3_ = 7.51, *P = 0.057*) and *Apis mellifera* (*X^2^*_3_ = 0.88, *P = 0.83*); the number of interactions performed by solitary bees (Fig. 4b, *X^2^*_3_ = 8.87, *P = 0.03*), Syrphidae (Fig. 4c, *X^2^*_3_ = 22.27_,_ *P<0.001*), Coleoptera *(X^2^*_3_ = 27.9_,_ *P<0.001*), other flies (*X^2^*_3_ = 24.02_,_ *P<0.001*), Lepidoptera (*X^2^*_3_ = 10.30, *P = 0.01*) and bugs (*X^2^*_3_ = 8.49 *P = 0.03*) was significantly impacted by the landscape context.

**Table 1:** Summary of post-hoc tests based on Tukey’s test for the number of interactions performed by insect morphotypes among the different landscape contexts.

	syrphidae		solitary bees		other flies	
	diff	p-value	diff	p-value	diff	p-value
semi-natural/agricultural	−0.29	0.82	−0.08	0.99	−0.14	0.99
semi-natural/suburban	1.71	<0.001	2.06	0.008	1.38	0.07
semi-natural/urban	0.96	0.03	1.03	0.37	2.94	<0.001
agricultural/suburban	2.06	<0.001	2.14	0.005	1.53	0.04
agricultural/urban	1.26	0.002	1.12	0.3	3.09	<0.001
suburban/urban	−0.8	0.14	−1.02	0.39	1.56	0.04
	**coleoptera**		**bugs**		**lepidoptera**	
	**diff**	**p-value**	**diff**	**p-value**	**diff**	**p-value**
semi-natural/agricultural	−0.16	0.98	−0.22	0.96	1.16	0.51
semi-natural/suburban	−1.22	0.06	−0.49	0.74	1.87	0.27
semi-natural/urban	3.14	<0.001	1.37	0.29	4.16	0.03
agricultural/suburban	1.38	0.02	−0.27	0.93	0.7	0.91
agricultural/urban	3.3	<0.001	1.59	0.16	2.99	0.2
suburban/urban	1.92	0.006	1.86	0.07	2.28	0.49
	**bumblebees**		**other hymenoptera**		***Apis mellifera***	
	**diff**	**p-value**	**diff**	**p-value**	**diff**	**p-value**
semi-natural/agricultural	0.52	0.65	1.84	1	−2.32	0.91
semi-natural/suburban	0.73	0.42	−4.72	0.9	−1.98	0.98
semi-natural/urban	0.6	0.53	1.84	1	−2.61	0.92
agricultural/suburban	0.21	0.97	−1.89	1	0.37	1
agricultural/urban	0.08	0.99	1.32	1	0.28	1

**Figure 5:**
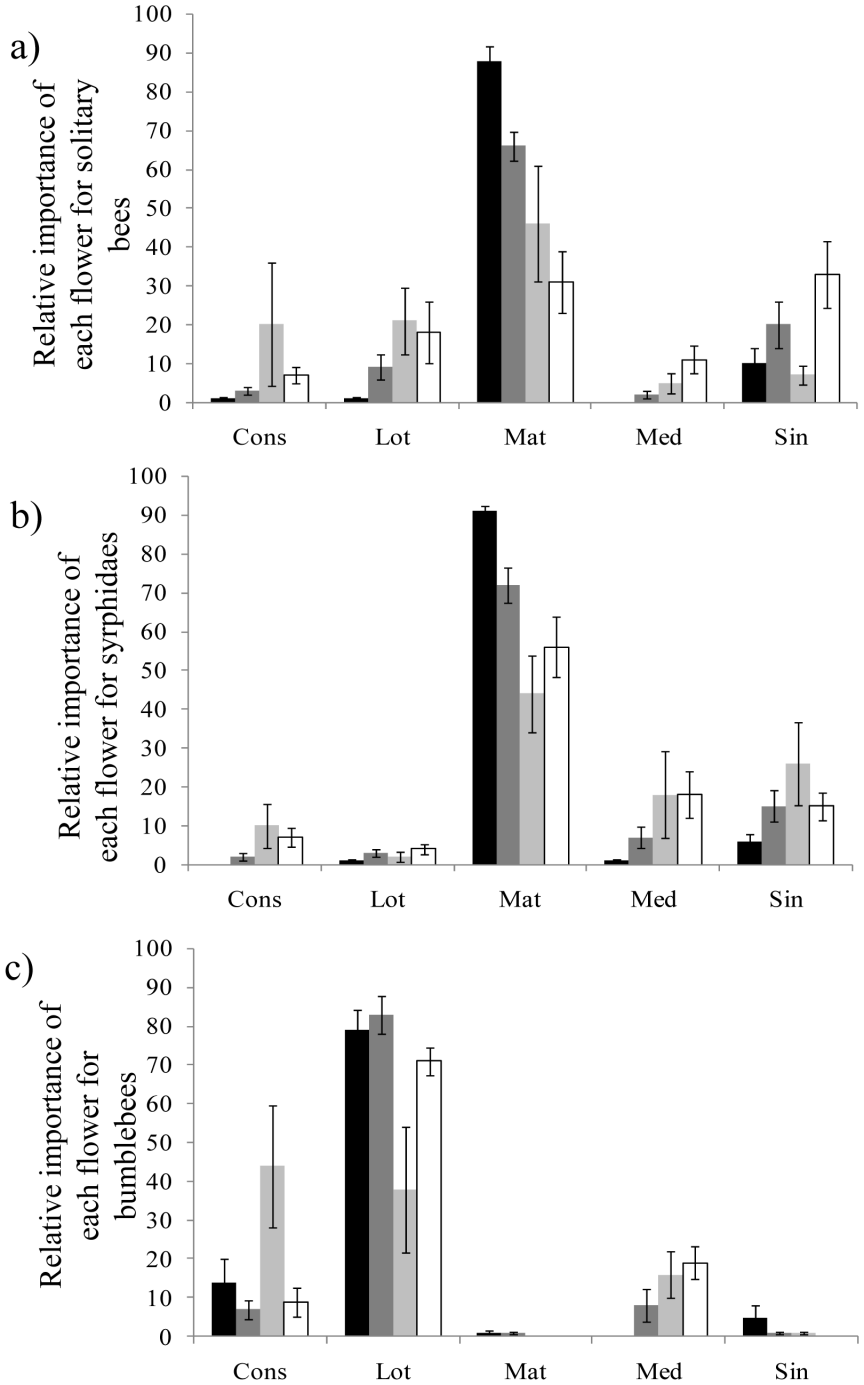
Mean number of visits per plant species for 3 pollinator morphotypes according to landscape contexts. Cons: *C. regalis*; Lot: *L. corniculatus*; Mat: *M.inodora*; Med: *M. sativa*. Sin: *S. arvensis*. Black bars: semi-natural context; dark grey bars: agricultural context; light grey bars: suburban context; white bars: urban context. Lines above bars denote 95% confidence interval.

Distribution of visits: We analysed the relative importance of each plant species for each flower visitor group in the different landscape contexts. The relative proportions of visits among flower species significantly differed among landscape contexts, in all flower visitor morphotypes : solitary bees (Fig. 5a; Pearson’s Chi-squared test: Chi^2^ = 116.39, d.f. = 12, *P<0.001*); Syrphidae (Fig. 5b; Pearson’s Chi-squared test: Chi^2^ = 66.56, d.f. = 12, *P<0.001*); bumblebees (Fig. 5c; Pearson’s Chi-squared test: Chi^2^ = 97.68, d.f. = 12, *P<0.001*); *Apis mellifera* (Pearson’s Chi-squared test: Chi^2^ = 186.18, d.f. = 12, *P<0.001*); Coleoptera (Pearson’s Chi-squared test: Chi^2^ = 56.03, d.f. = 12, *P<0.001*); Lepidoptera (Pearson’s Chi-squared test: Chi^2^ = 175.39, d.f. = 12, *P<0.001*); and other flies (Pearson’s Chi-squared test: Chi^2^ = 67.04, d.f. = 12, *P<0.001*).

**Figure 6:**
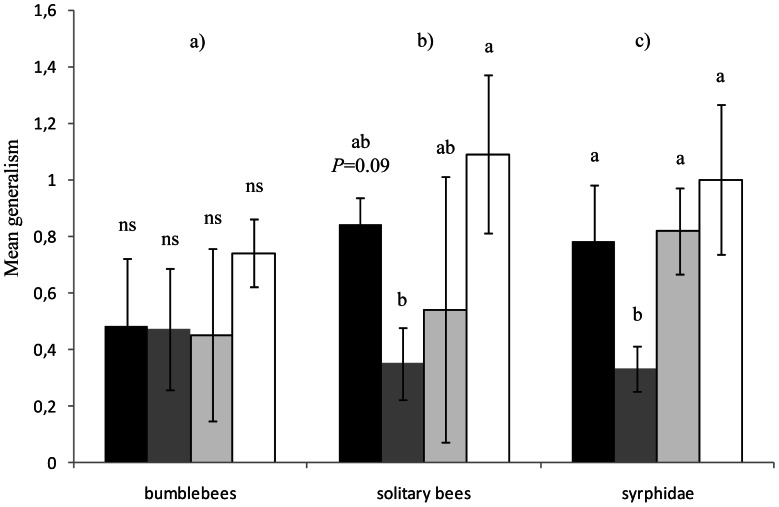
Mean weighted generalism for 3 pollinator morphotypes according to the landscape context. a) bumblebees. b) solitary bees. c) Syrphidae. Black bars: semi-natural context; dark grey bars: agricultural context; light grey bars: suburban context; white bars: urban context. Lines above bars denote 95% confidence interval. Bars that do not share the same letter show significant differences (P<0.05). NS: no significant differences (P>0.05).

Flower visitor generalism: Changes in the distribution of visits made by flower visitors between the different plants species were in some cases associated with changes in their generalism degree: these results are presented in Fig. 6 and Table 2. The effect of the urbanisation gradient on generalism depended upon the flower-visitor morphotype considered (significant interaction between insect morphotypes and landscape context (*X^2^*_24_ = 72.31, *P<0.001*). While weighted generalism of bumblebees (Fig. 6a; *X^2^*_3_ = 3.83, *P = 0.27*); Lepidoptera (*X^2^*_3_ = 5.67, *P = 0.12*); *Apis mellifera* (*X^2^*_3_ = 3.35 *P = 0.34*) and other Hymenoptera (*X^2^*_3_ = 3.02, *P = 0.38*) did not change regarding the landscape context; the weighted generalism of solitary bees (Fig. 6b; *X^2^*_3_ = 8.78, *P = 0.03*), Syrphidae (Fig. 6c; *X^2^*_3_ = 12.95, *P = 0.004*) andother flies (*X^2^*_3,18_ = 8.54, *P = 0.03*) was modified by the landscape context.

**Table 2:** Summary of post-hoc tests based on Tukey’s test for the generalism degree of insect morphotypes among the different landscape contexts.

	syrphidae		solitary bees		other flies	
	diff	p-value	diff	p-value	diff	p-value
semi-natural/agricultural	0.46	0.01	0.5	0.09	0.04	0.98
semi-natural/suburban	−0.1	0.9	0.19	0.83	−0.38	0.018
semi-natural/urban	−0.22	0.43	−0.26	0.62	−0.09	0.87
agricultural/suburban	−0.56	0.002	−0.3	0.54	−0.43	0.007
agricultural/urban	−0.68	<0.001	−0.76	0.002	−0.13	0.69
suburban/urban	−0.12	0.86	−0.45	0.18	0.29	0.11
	**coleoptera**		**bugs**		**other hymenoptera**	
	**diff**	**p-value**	**diff**	**p-value**	**diff**	**p-value**
semi-natural/agricultural	0.07	0.98	NS	NS	0.07	0.98
semi-natural/suburban	0.14	0.92	NS	NS	−0.27	0.57
semi-natural/urban	0.35	0.39	NS	NS	0.07	0.98
agricultural/suburban	0.06	0.99	NS	NS	−0.34	0.36
agricultural/urban	0.27	0.61	NS	NS	0	1
suburban/urban	0.2	0.81	NS	NS	0.34	0.57
	**bumblebees**		**lepidoptera**		***Apis mellifera***	
	**diff**	**p-value**	**diff**	**p-value**	**diff**	**p-value**
semi-natural/agricultural	0.01	1	0.32	0.22	0	1
semi-natural/suburban	−0.1	0.92	0.23	0.55	0	1
semi-natural/urban	−0.27	0.27	0.4	0.07	0.1	0.41
agricultural/suburban	−0.11	0.89	−0.08	0.96	0	1
agricultural/urban	−0.28	0.23	0.08	0.95	−0.1	0.41
suburban/urban	−0.17	0.69	0.17	0.55	−0.11	0.43

#### Plant functional groups

**Figure 7:**
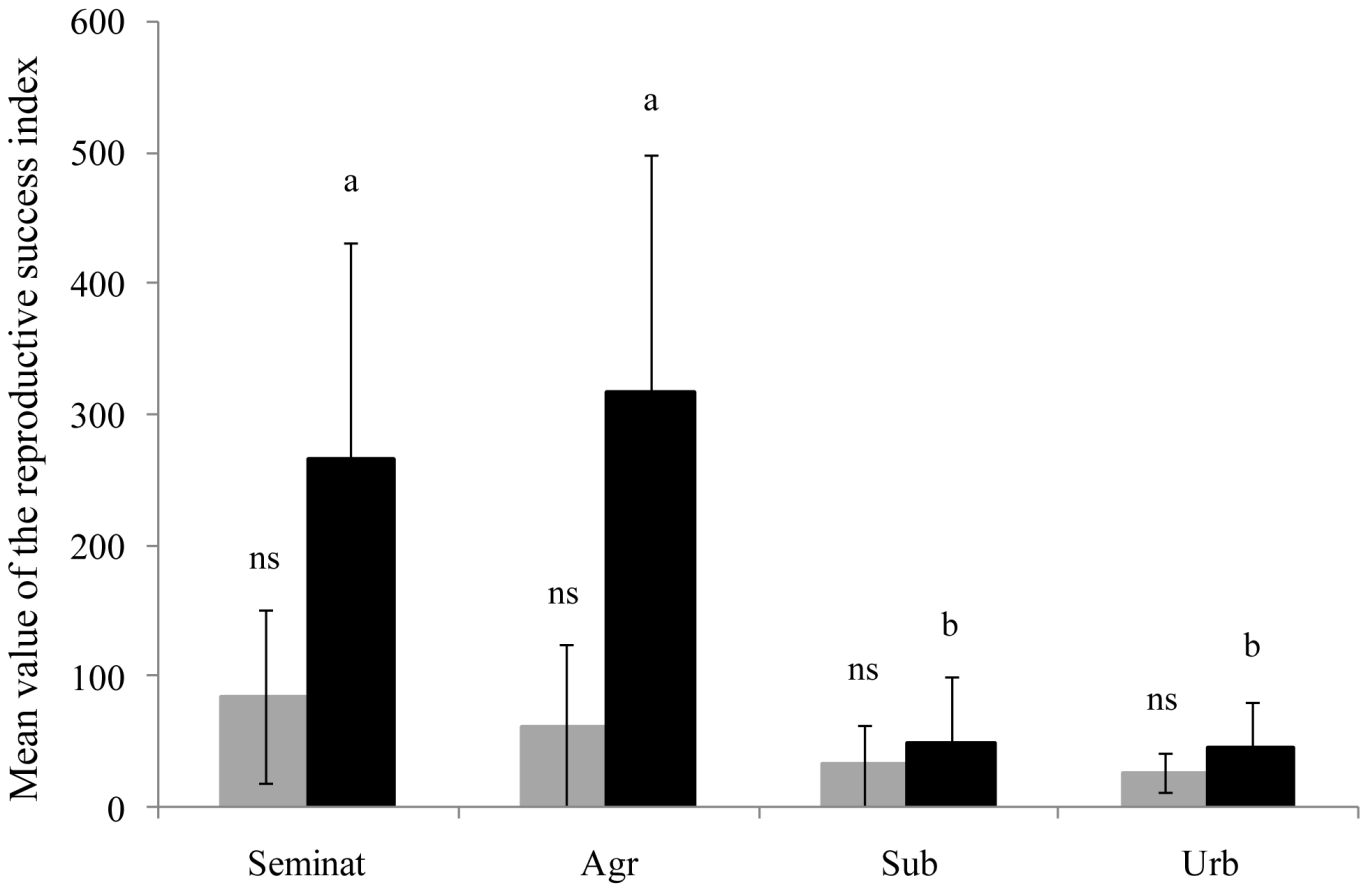
Mean reproductive success index of plant morphological groups according to the landscape context. Black bars: open flowers morphological group. Grey bars: tubular flowers morphological group. Lines above bars denote 95% confidence interval. Bars that do not share the same letter show significant differences (P<0.05). NS: no significant differences (P>0.05). Seminat: Semi-natural; Agr: Agricultural; Sub: Suburban; Urb: Urban.

The effect of landscape context on estimated reproductive success index of plants depended on the plant functional group (Fig. 7; significant interaction between plant functional group and landscape context *X^2^_3_* = 13.93, *P = 0.002*). The mean reproductive success index of plants in the open flower group was affected by the landscape context (*X^2^*_3_ = 14.04, *P = 0.002*). Open flowers had a significantly higher reproductive success index in agricultural landscape context than in suburban (Tukey test *P = 0.002*) and urban (Tukey test *P = 0.002*) areas. In the same way, open flowers had a significantly higher reproductive success index in semi-natural landscape context than in suburban (Tukey test *P = 0.003*) and urban (Tukey test *P = 0.002*) areas. The reproductive success of the tubular flower group was not significantly impacted by landscape context (*X^2^*_3_ = 3.91, *P = 0.271*).

## Discussion

In this study, we used an experimental approach to analyse how increasing levels of urbanisation had an impact on plant-pollinator interaction webs. Controlling for the composition of plant community enabled us to understand how observed modifications in pollinator communities along an urbanisation gradient affected the structure and functioning of the pollination network. Our results clearly show that urbanisation not only negatively affected the number of interactions between pollinators and the experimental plant community, but also the diversity of these interactions. The visitation frequency of Syrphidae, other flies, solitary bees and Coleoptera was significantly lowered by increasing urbanisation, whereas visitation frequency of bumblebees and *Apis mellifera* (able to forage on all floral morphologies) remained the same across the urbanisation gradient. These variations in visitation frequencies were linked with changes in pollinator generalism, with increased generalism for Syrphidae, solitary bees and other flies along the urbanisation gradient, whereas the generalism degree of bumblebees and *Apis mellifera* did not vary. These changes were reflected at the network level by higher interaction evenness in urban plant-pollinator webs. Overall, these modifications had important consequences for the ecology of the experimental plant community, with a lower reproductive success index in urban environments for plants belonging to the open flower morphological group.

In what follows we will discuss our results within the framework of the links between urbanisation and the functioning of plant-pollinator networks and address the limitations of our approach.

### Flower-visitor Morphological Groups along Urbanisation Gradient

Biesmeijer et al. [13] already noted that pollinators with narrow habitat requirement *i.e.* specialist species with short mouthparts and limited flight distance, tended to decline more than generalist species. Our study is consistent with these results. We showed that the number of interactions performed by solitary bees and Syrphidae (55% of whole interactions) decreased in urban and suburban environments compared to semi-natural and agricultural ones. These groups are in majority composed of small species with most of individuals belonging to the Halictini tribe for solitary bees, and a majority of *Episyrphus balteatus* for Syrphidae (see Supporting Information S2). Body size and mouthparts length of pollinating insects are correlated traits [45], insects with smaller body sizes having on average smaller mouthparts than insects with larger body sizes. Flower visitors with short mouthparts are usually considered as specialists for foraging on open-corolla flowers and might thus exploit only a restricted range of resources. Furthermore, flower visitors with smaller body sizes often present lower mobility [16]. Flight ability limits foraging range and the capacity to switch between rewarding patches [46]. Consequently, small pollinators are often more impacted by perturbation [47], [48] and more sensitive to habitat fragmentation and increasing urbanisation [8], [9], [16]. In contrast, morphotypes such as bumblebees and *Apis mellifera* were not impacted by the landscape context. Bumblebees for example have been shown to be weakly impacted by habitat fragmentation ([49]; see also [50]) as induced by urbanisation ([8]; but see [4]), probably because of their ability to fly over long distances and forage between patches of suitable habitats [51], [52]. Moreover, as shown by Fontaine et al. [12] bumblebees are able to visit open flowers and tubular flowers and are considered as generalist pollinators. This ability to visit all flower morphologies might allow them to exploit more resources than specialist foragers and might prevent them from the negative effect of urbanisation.

We also showed that all pollinator morphological groups modified their diet composition along the urbanisation gradient. Overall, the distribution of visits between the different plant species was more balanced in suburban and urban landscape contexts. However, flower-visitors such as bumblebees mostly visited the same three plant species (*C. regalis, L. corniculatus, M. sativa*) all belonging to the tubular morphological group, independently of the landscape context. Bumblebees are reported as constant foragers [36] and they often limit their visits to few species [53]. Also, because of the length of their mouthparts, pollinators such as bumblebees preferentially forage on plants best adapted to their morphology with elongated tubular corollas [12], [54]. On the contrary, small pollinators (Syrphidae, other flies and solitary bees) tended to distribute their visits more equitably between the different plant species within suburban and urban landscape contexts, showing increased generalism along the urbanisation gradient (see Fig. 5; Fig. 6). Several hypotheses can explain these differences in generalism along the urbanisation gradient: 1) change in the composition of pollinator community toward a dominance of species with broader ecological niches, as reported by Zanette *et al.* [6] (see also [55] for examples on Coleoptera) and/or 2) change in foraging behaviour of some species. In urban environments, it has been reported that plant species with open flowers easily accessible to pollinators with short mouthparts could be less abundant (Cane, 2005 in [10]), leading to increased competition for these resources in urbanised environments and ultimately to an increase in diet breadth of these pollinators. To avoid competition, flower-visitors might change their behaviour toward a wider variety of plants and increase their generalism degree ([56]; but see [57]) whereas at high flowering density and richness, flower-visitors tend to specialise [58], [59]. As summarised by Kunin & Iwasa [60]: “Where floral resources are scarce, pollinators should behave as generalists whereas when resources are superabundant, specialization on the single most profitable flower type (all else being equal, the commonest one) is favoured”. However, these results require further investigation to better understand if changes of generalism level in urbanised habitats are solely induced by modifications in the composition of pollinator communities, or if pollinator species lower their acceptance threshold in an urbanised context.

Results also show that Syrphidae were more generalist in semi-natural landscape context than in agricultural one. Such resource specialisations in agricultural habitats have been seen in host-parasitoids interaction networks: Tylianakis *et al.* recorded [32] that a species able to parasite 16 host species focused the great majority of attacks on the commonest host in an agricultural context. In agricultural landscapes, pollinators are often confronted to mass flowering crops [61], with resources available in large quantities, this could select exploitative use of flowers by pollinators (high floral constancy). Another possible explanation lies in the different spatial complexity between complex semi-natural and simplified agricultural landscapes. Laliberté & Tylianakis [62] underlined that habitat complexity could affect foraging abilities among parasitoids. We argue here that these pollinators might be less selective when foraging in semi-natural conditions where spatial patchiness in plant distribution is common [63]. However, this hypothesis requires further investigation.

### Changes in Network Structure along the Urbanisation Gradient

Along the urbanisation gradient, we recorded a lower number of interactions between our plant community and pollinator morphotypes such as Syrphidae and solitary bees, together with an increased generalism degree of these groups. This directly impacted the structure of the plant-pollinator networks. We observed a low evenness in agricultural landscape context, and interaction evenness is low when pollinators strongly interact with few plant species [64]. This is consistent with the increased specialization of pollinator groups such as Syrphidae we observed in agricultural context. In urban networks, interaction evenness was high, which could be linked to higher generalism of solitary bees, other flies, and Syrphidae. It has been suggested that higher interaction evenness could be associated with overall sustainability of the plant-pollinator community [11]. However, this is rather unlikely here, as we recorded fewer interactions in this same context. Our results also underline the importance of taking into account quantitative data when analysing the structure of interaction networks [65], [66]. Analysing solely the number of links did not enable us to detect any changes in network structure along our urbanisation gradient. In the literature, weighted indices such as interaction evenness are reported as good estimators of changes in networks structure (see [32]) especially with approaches integrating pollinator behaviour (see [67]).

### Reproductive Success of the Experimental Plant Community along the Urbanisation Gradient

Along the urbanisation gradient, we recorded a significant decrease in the number of interactions carried out by morphotypes which mostly visited flowers with easily accessible rewards (i.e. morphotypes with relatively smaller mouthparts). As a consequence, we observed a significant decrease in the reproductive success index of open flowers morphological group, in the suburban and urban habitats. Mutualistic networks being asymmetrical, the recorded loss of interactions performed by flower visitors might be buffered by interactions with generalist long mouthparts foragers [67], [68]. However our data show that flower visitors with longer mouthparts such as bumblebees tended to focus mainly on tubular flowers, in all landscape contexts along our urbanisation gradient. Bumblebees are known to forage mainly on species best adapted to their morphology [69]. Moreover, visits performed by generalist pollinator species are often less effective than visits made by specialist pollinator species [69]. In general, a higher level of specialization leads to better pollination success due to higher flower constancy and higher quantity of pollen removal and pollen transfer [70]–[72]. The increase in generalism of open flower-visitor insects might thus also contribute to the predicted decrease in the reproductive success of plant this group. Here, calculation of our reproductive success index was based on the number of visitation events that might affect the reproductive success of a plant species. In this case we consider that a low amount of conspecific pollen deposited on stigmas of a given plant species translates into a low reproductive index. Pollen limitation is acknowledged as one of the principal causes of reduction of plant reproductive success in fragmented habitats [73]. In our experiment, low reproductive index for open-flowers morphological group may indicate to what extends the potential reproductive success of this group could be affected by urbanisation.

To our knowledge, this is the first experimental approach to analyze the impact of urbanisation on plants and pollinators with a particular focus on their interactions and the resulting structure of the plant-pollinator network. Moreover, we believe than controlling for the composition of the plant community is a powerful tool to study plant-pollinator interactions. However, our approach still has several key limitations that will need to be addressed in future studies. First, the lack of identification to the species level for insects calls for caution concerning the generalisation of our conclusions. Particularly, we do not know whether observed changes in network structure along the urbanisation gradient are due to changes in species composition within flower visitor morphotypes or to changes in species foraging behaviour in these morphotypes. Building networks at species level along urbanisation gradients should greatly improve our knowledge on this research field. Second, we estimated plant reproductive success by developing an index based on observed interaction frequencies within plant-pollinator networks. However, future studies will need to precisely estimate the reproductive success of plant communities by directly measuring fruit set or seed set of plant communities. Our new index will also need to be tested against observed measures of plant reproductive success. Finally, plant-pollinator networks show great variability over time [15], [74], and in order to built networks with an overview on all possible interactions, future studies should be carried out on longer periods of time.

Overall, our findings suggest that both short mouthparts pollinators and open flower plant species seem especially sensitive to increasing urbanisation. In a context of ever-increasing impact of urban areas on natural habitats, our results shed a light on possible conservation recommendations concerning plants and pollinators. Conservation practices aiming at preserving the functionality of plant-pollinator networks should promote the maintenance of both specialist flower-visitor groups and open flower plant species. However, in many urban environments, actual conservation measures are mostly focused on a single generalist pollinator species, the domesticated honeybee *Apis mellifera.* For example, in the city of Paris (France), close to 300 hives have been established over the past few years [75]. The importance of honey bees in conservation measures has led to a recent controversy ([76], [77] see also [78]) and in the light of our results we feel that these reintroduction programs should be carried out with caution, because of their potential negative impact on specialist pollinators that already are threatened in urban environments.

## Supporting Information

Supporting Information S1 **Further information and permits for field sites.**
(DOCX)

Supporting Information S2 **Precisions on flower-visitor morphotypes.**
(DOCX)
