# Supplementary material for: Plant Pollinator Networks along a Gradient of Urbanisation
Source: PLoS One. 2013 May 22;8(5):e63421. doi: 10.1371/journal.pone.0063421 (PMC3661593; doi:10.1371/journal.pone.0063421)
Supplement: Supporting Information S1 — Further information and permits for field sites. (DOCX) [file pone.0063421.s001.docx]

**S1** Further information and permits for field sites

All necessary permits were obtained for the field sites described below.

a) For semi-natural sites: 1) Plateau de Foljuif (permission obtained from the CEREEP Ecotron IleDeFrance; GPS coordinates: 48°16'54.84"/2°39'58.88"). 2) Prairie de St Lambert (permission obtained from Alexandre Mari, Parc Naturel Régional de la Haute Vallée de Chevreuse; GPS coordinates: 48°44'16.28"/2°01'05.66"). 3) La Mulasserie (permission obtained from Christophe Montagnier UE 1246 INRA; GPS coordinates: 48°48'20.74"/2°05'39.14").

b) For agricultural sites: 1) Bezanleu (permission obtained from Isabelle Jouanneau CEREEP Ecotron IleDeFrance; GPS coordinates: 48°17'07.23"/2°48'45.24"). 2) Le Grand Ambézys (permission obtained from the Parc Naturel Régional de la Haute Vallée de Chevreuse; GPS coordinates: 48°43'44.36"/1°58'22.54"). 3) Plateau de Grignon (permission obtained from Christophe Montagnier UE 1246 INRA; GPS coordinates : 48°50'29.60" /1°56'24.92").

c) For suburban sites: 1) Mairie de Nemours (permission obtained from the CEREEP Ecotron IleDeFrance ; GPS coordinates: 48°15'54.70"/2°41'09.80"). 2) Cimetière de Versailles (permission obtained from Cathy Biass-Morin, Mairie de Versailles; GPS coordinates: 48°47'27.00" /2°08'21.20"). 3) Mairie de Bonnelles (permission obtained from the Parc Naturel Régional de la Haute Vallée de Chevreuse; GPS coordinates: 48°37'07.21"/2°01'43.59").

d) Urban sites : 1) Jardin Ecologique du Jardin des Plantes (permission obtained from Philippe Barré ; GPS coordinates : 48°50'38.13" /2°21'40.54"). 2) Jardin de l’Ecole des Mines (permission obtained from Dominique Guiraud-Deville; GPS coordinates: 48°50'41.93"/2°20'18.94"). 3) Parc de la Cité Internationale Universitaire de Paris (permission obtained from David Otamendi; GPS coordinates: 48°49'13.90" /2°19'52.03").
