# Supplementary material for: Plant Pollinator Networks along a Gradient of Urbanisation
Source: PLoS One. 2013 May 22;8(5):e63421. doi: 10.1371/journal.pone.0063421 (PMC3661593; doi:10.1371/journal.pone.0063421)
Supplement: Supporting Information S2 — Precisions on flower-visitor morphotypes. (DOCX) [file pone.0063421.s002.docx]

**S2** Precisions on flower-visitor morphotypes.

In parallel to this experimentation, we made insect captures using coloured pan-traps near our field sites. We captured more than 2 000 insects and identified them to the species level. The results of this second study (Geslin et al., manuscript in preparation) give us some indications on the composition of insect functional morphotypes:

a) Bumblebees: Because of the difficulty to visually discriminate between Bombus and the parasitic sub-genus Psithyrus and some other species such as their hosts, some bumblebees from this genus might be included in our morphotype “Bombus”. However, a single individual belonging to the sub-genus Psithyrus (*Bombus sylvestris*) has been captured in our pan traps. Thus, even though we are aware that this genus does not participate to the pollination of plants because of its inability to collect pollen grains, we considered their presence as anecdotal in our data.

b) Solitary bees: the solitary bees group included all individuals from Apoidea except those from genus *Bombus* and *Sphecidae* and except *Apis mellifera*. More specifically, this gathers individuals from Andrenidae, Colletidae, Halictidae, Melittidae, Apidae, Dasypodaidae and Megachilidae families. *Sensus stricto*, in our pan traps we found individuals from Halictus, Lassioglossum, (more than 75% of whole solitary bees for these two genera), Chelostoma, Andrena, Megachile, Heriades, Osmia, Colletes, Dasypoda and Hylaeus.

c) Syrphidae: Syrphidae regroups individuals from Syrphus, Trichopsomyia, Sphaerophoria, Scaeva, Melanostoma, Eupeodes, Eristalis and Episyrphus (*Episyrphus balteatus* represented 77% of the whole Syrphidae morphotype).

d) Other Hymenoptera: this group comprised aculeate wasp from the Vespoidea super-family, Vespidae family and mostly from Vespula and Dolichovespula genera, even though individuals from Vespa and Polistes genera might also be comprised in this group.
